# Applications of Computational Fluid Dynamics in Congenital Heart Disease: A Review

**DOI:** 10.3390/jcdd12020070

**Published:** 2025-02-13

**Authors:** Amartya Dave, Raquel dos Santos, Usmaan Siddiqi, Aashi Dharia, Willa Li, Umar Siddiqi, Nhung Nguyen, Luka Pocivavsek, Narutoshi Hibino

**Affiliations:** 1 Section of Cardiac Surgery, Department of Surgery, The University of Chicago, Chicago, IL 60637, USA; amartya.dave@ucsf.edu (A.D.);; 2 School of Medicine, University of California, San Francisco, CA 94143, USA; 3 Department of Surgery, University of California, San Francisco, CA 94143, USA; 4 Section of Vascular Surgery, Department of Surgery, The University of Chicago, Chicago, IL 60637, USAlpocivavsek@bsd.uchicago.edu (L.P.); 5 Pediatric Cardiovascular Surgery, Heart Institute, Advocate Children’s Hospital, Oak Lawn, IL 60453, USA

**Keywords:** computational fluid dynamics, congenital heart disease, Fontan, aortic coarctation

## Abstract

Computational fluid dynamics (CFD) is a tool that allows for the analysis of otherwise unobservable blood flow patterns. In the context of medicine, CFD enables researchers to better understand acute and chronic pathophysiology as well as utilize modeling tools to predict blood flow patterns in response to surgical intervention. Such a tool is particularly useful in the field of congenital heart disease (CHD), where complex geometries and patient-specific pathology are common. Research applying CFD to study CHDs has significantly grown in the last twenty years, with new methodologies and recommendations being published at an even faster pace in the last decade. Many currently available reviews are focused on a particular area of progress or on the technical approaches to CFD geared toward the clinician. This review focuses on CFD application within the major domains of CHD research, specifically single ventricle defects and aortic coarctation, reviewing consensus seminal work while highlighting more recent avenues of study. Balancing discussion of CFD parameters with potential clinical implications of study results, this review not only aims to provide cardiovascular professionals context for the technical advancements being made in the field but also a sense of contemporary CFD’s utility in clinical practice.

## 1. Introduction

Congenital heart disease (CHD) encompasses a spectrum of morphologies that impair normal blood flow in the heart. In recent decades, advancements in the field have helped patients with CHD live well into adulthood. Despite this, CHD’s long-term morbidity and mortality have increased in the third and fourth decades of life [1,2]. Abnormal blood flow patterns that last long after surgical or endovascular repair may impact long-term outcomes by increasing the chances of hospitalization and repeated intervention [3]. As such, a key question arises: how can flow be accurately characterized and subsequently optimized for CHD repair? In practice, this question is directly applicable to operations in pediatric cardiac surgery—graft or shunt configuration and size, ideal artery diameters, ideal pulmonary flow, and many other crucial parameters can be altered to potentially optimize blood flow. To begin to answer these questions, the seminal work of de Leval and colleagues in 1996 offered computational fluid dynamics (CFD), an engineering field that uses computer simulation to study the behavior of fluid flows, as a numerical method to objectively understand cardiovascular flow phenomena [4]. This advancement introduced the concept of a team-based approach to better characterize complex congenital heart diseases, one that includes both cardiac surgeons and engineering specialists. Through such a collaboration, the idea of optimizing surgical interventions on a patient-specific basis has been made possible. Among many other applications that will be discussed, a CFD team can pre-operatively compute hemodynamic changes from simulated surgical manipulation and predict post-operative flow patterns to ultimately suggest changes to reduce deleterious alterations that may cause long-term clinical complications. In this review, we aim to provide the reader with a synopsis of what CFD work entails for CHD morphologies and how recent studies have supplemented pioneering work to further the understanding of CHD.

## 2. What Is CFD?

In industry, CFD serves as a tool for optimizing product performance, refining design processes, and constructing precise models of fluid behavior under diverse conditions. These advancements facilitate greater efficiency, cost reduction, and sustainable resource utilization across sectors such as aerospace, automotive engineering, and energy production. While the study of fluids at rest (hydrostatics) and fluids in motion (fluid dynamics) both fall under the broader field of fluid mechanics, CFD is primarily employed to analyze dynamic fluid behavior. This includes steady-state flows, where the flow field varies spatially but remains constant over time, as well as unsteady flows, where the flow field changes both spatially and temporally [5]. In a general sense, the core of CFD simulations is mathematical modeling and solving to better understand how fluid flows may behave under various conditions. Whether it is to minimize drag in aircraft, design effective cooling systems for engines and batteries in electric vehicles, optimize emissions from power plants, or reduce long-term complications from complex heart surgery, CFD has allowed for the study of improving systems in our everyday world that run on dynamic fluid flows.

With improving cardiac imaging technology and clinical investigation, it is now possible to use CFD techniques to construct and analyze patient-specific models of conditions ranging from pediatric congenital heart disease (CHD) to vascular aneurysms and even everyday stresses such as exercise. By pairing cardiac imaging modalities such as magnetic resonance imaging (MRI), ultrasound, and cardiac computed tomography (CT) with CFD simulation, researchers can characterize fluids by calculating basic values such as pressure, velocity, and flow rate and otherwise immeasurable ones such as energy loss and wall shear stress (WSS). These terms are defined in Table 1. As will be discussed later in this review, energy loss and WSS are critical parameters that CFD researchers utilize to optimize fluid flow in Fontan patients. Broadly speaking, energy loss correlates to the efficiency of the circulation whereas WSS allows for analysis of stress patterns along the vessel wall, helping predict areas of stasis or abnormally high values that may induce endothelial injury.

No CFD simulation is completed with the click of a button. In fact, the creation of a physiologically representative and computationally feasible model is a thorough process generally broken down into three distinct workflow phases: pre-processing, simulation, and post-processing. Although this description of CFD workflow is generally applicable to many studies, various technical methods may be altered depending on the desired outcome and improvements in numerical analysis.

## 3. Traditional CFD Workflow

### 3.1. Pre-Processing: From Clinic to Computer

The first step in the CFD process is to utilize cardiac imaging data (usually in the form of MRI or CT) to reconstruct a three-dimensional model representative of the patient’s anatomy. Many times, researchers will define a “region of interest” within the imaging data to focus the CFD calculation. Since CFD simulation is a computationally expensive process, a region of interest serves to avoid spending resources on areas in which simulation is not needed. Once this is completed, the medical imaging data must be converted into a corresponding digital geometric model—this process is termed “segmentation”. Segmentation produces a model that includes either only geometry or both geometry and density (determined using pixel grayscale values). This process can be performed through 2D segmentation, 3D segmentation, or shape-based modeling [6,7]. 3D segmentation is largely preferred because accurate simulation of three-dimensional fluid dynamics requires a corresponding 3D geometry. Programs such as ‘Mimics’ or ‘Simpleware ScanIP’ facilitate the creation of these models by reconstructing imaging data through intensity thresholding, where image intensity defines the relative location of lumen boundaries [7]. Note that although such programs can enable automatic segmentation of medical imaging, manual manipulation during segmentation is usually required to improve the accuracy of the model. Many times, variable contrast in medical imaging can introduce errors into the segmentation. Thus, although tedious, it may be necessary for the CFD team to work with the surgeon to ensure that segmentation is being performed accurately on the appropriate anatomy.

After converting imaging data into a 3D model, an essential step follows—“mesh discretization”—which is performed using a numerical method such as the element method (FEM). In this process, the flow domain of the smooth 3D model is divided into a large number of small elements, or “finite elements”. This is conducted by marking numerous nodes (typically on the order of 10^5^ to 10^6^) throughout the geometry. Each finite element represents a discrete section of a complex geometry that is small enough to allow the governing equations of fluid flow to be solved over it [8]. Since these coupled partial differential equations are too complex to be solved analytically for an entire domain, the finite element method (FEM) enables a numerical solution by breaking the geometry into smaller, more manageable elements. The individual solutions for each element are then combined to approximate the solution for the entire computational domain. To assess the accuracy of the results, researchers perform “mesh convergence” studies, in which the mesh is refined by progressively reducing the element size. A finer mesh generally leads to more accurate results, as it more closely represents the complex geometry, similar to how increasing the number of line segments used to approximate a circle results in a shape that better resembles the true curve. However, finer meshes come at the cost of increased computational time and resource usage, requiring a balance between accuracy and efficiency.

### 3.2. Simulation: Solving the Fluid Problem

The Navier–Stokes equations form the foundational governing equations for fluid dynamics and are essential for CFD simulations. These equations describe the motion of fluids by relating density, velocity, pressure, viscosity, and external force. Note that blood density and blood viscosity are assumed constant and typically never truly measured in practice. Solving the Navier–Stokes equations for complex geometries, such as the Fontan pathway, allows researchers to obtain critical flow characteristics, as described in Table 1. Solving the Navier–Stokes equations is performed iteratively through a computational algorithm. There are a number of solution methods, many of which will be mentioned further in this text.

Typically, the fluid flow within the region of interest involves at least one inlet and one outlet. One-dimensional (1D) models are often utilized for flow occurring in veins and arteries by averaging incompressible Navier–Stokes equations over a vessel cross-section. Zero-dimensional (0D) models, or lumped-parameter models, are often utilized for flow occurring in arterioles, capillaries, and venules and are obtained by averaging the 1D model over a vessel’s length while making some assumptions [9]. CFD solvers require the specification of physiological parameters at each boundary of the computational domain (e.g., the walls of blood vessels or the inflow/outflow regions) at all times. It is important to note that the establishment of these parameters, or “boundary conditions”, is significant due to their large role in determining simulation results and linking clinical data to computed fluid flow. Generally, boundary conditions take the form of clinically measured velocity profiles or pressures at the inlet and outlet of the region. At times, obtaining these clinical measurements can be difficult, especially if there are constraints regarding invasiveness. Currently, routine MRI and Doppler ultrasound are among the most commonly used modalities to obtain velocity measurements and anatomical information for CFD input. Cardiac catheterization, although invasive, is often the only option to obtain pressure waveforms. According to the literature, it is advised that catheterized regions be monitored across approximately 100–250 full cardiac cycles, depending on the patient, to achieve accurate results for simulation [10]. Due to clinical variation and hard-to-achieve precision, the generation of appropriate boundary conditions in CFD study remains one of the most challenging barriers in the field [11].

Once boundary conditions have been established and fluid-flow variables have been solved, the values in Table 1 can be evaluated. Usually, this CFD process will be conducted in pre- and post-intervention simulations to better tailor the procedure for optimal flow.

### 3.3. Post-Processing: Visualizing Results

After producing numerically dense results in simulation, post-processing is required to isolate and display data that are of specific interest. There are many post-processing data visualization techniques. Values such as WSS and Energy Loss can be colorized and graphed; 3D or 2D surface plots, contour plots, vector plots, and cardiac-cycle time plots are among the most utilized [12]. Post-processing visualization can include flow domain, boundary conditions, and CFD simulation results in tables and colorized models. Path lines offer a valuable addition to such plots, as they can help visualize virtual particle paths through the velocity field. After this visualization, the surgeon can supplement their clinical decision-making with understandable data.

**Figure 1:**
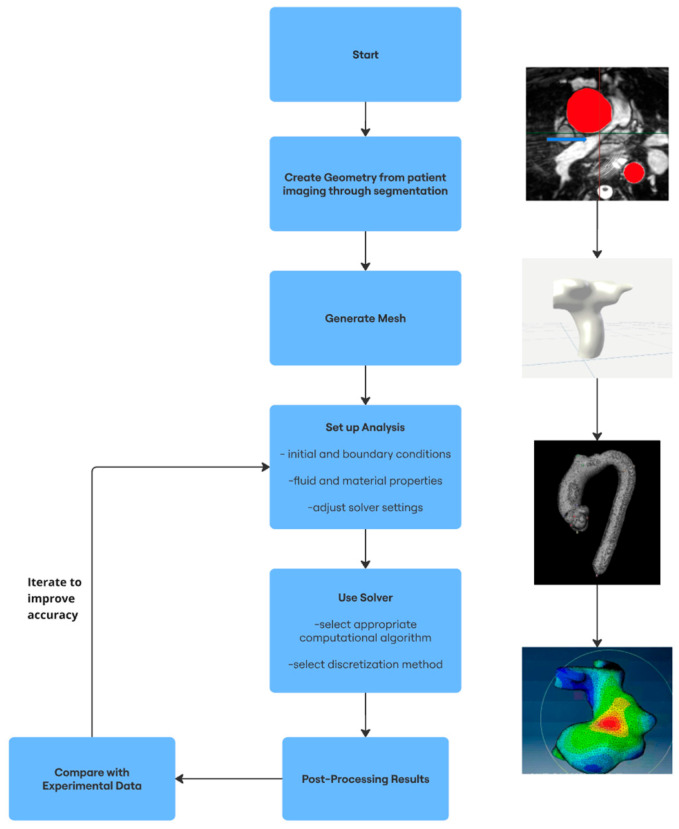
Summary of Typical CFD Workflow (Pre-Processing, Simulation, Post-Processing).

Figure 1 below is a brief visual summary of the discussed CFD workflow. Images demonstrate segmentation, geometry creation, mesh generation, and simulation, respectively.

## 4. CFD in Congenital Heart Disease

### 4.1. Fontan Surgery

#### 4.1.1. Introduction to CFD for Fontan Surgery

Patients with ventricular dysfunction, such as hypoplastic left heart syndrome (HLHS), tricuspid atresia, and double outlet right ventricle, will often require a combination of a three-stage procedure (Norwood, Glenn/Hemi-Fontan, and complete Fontan/total cavopulmonary reconstruction) that works to reroute systemic venous blood directly to the lungs past the non-functional ventricle. These interventions lead to varying circulatory hemodynamics, such as inadequate blood distribution and energy losses, that may be associated with long-term complications [13].

CFD has allowed the possibility of modeling the circulation of each of the three stages, providing data on how flow measures can change with alterations to patient vessel geometry. Since the work of de Leval in 1996, CFD research has grown tremendously in supporting decision-making, optimization of surgery, and evaluating outcomes for patients who undergo Fontan surgery. In the context of univentricular flow, early CFD studies focused on localized 3D computational or in vitro models that provided detailed descriptions of local hemodynamics but failed to describe interactions with global circulation because of fixed boundary conditions [14]. Despite this, earlier models contributed heavily to the optimization of the cavopulmonary connection and indicated that reduced energy loss is a key index in reducing deleterious fluid flow in Fontan patients. As technology evolved, models that utilized magnetic resonance velocity data began to add crucial factors such as vessel elasticity and ventilation effects to their computation. The original optimization scheme from de Leval [4] was recalculated by Hsia et al. [15], finding that a 19–20 mm conduit to the left of the superior caval vein minimizes power loss and balances pulmonary flow. Marsden and colleagues furthered this scheme by introducing the benefits of the Y-graft conduit, and Whitehead et al. applied these findings to exercise, discovering a non-linear relationship between flow rate and power dissipation when using resting MRI data and modeled exercise scenarios [16,17]. To better account for interactions with the rest of the circulatory system, a 0D, lumped parameter model (a non-linear model) that utilized previous simulation data to vary shunt resistance with shunt diameter and flow rate was developed. However, despite reasonable clinical alignment, parameter identification was challenging due to limited clinical data. Soon, the need to utilize the advantages of multiple models was realized, marked by Quarteroni and Veneziani’s description of one of the first heterogeneous models that coupled local three-dimensional blood flow with a systemic, zero-dimensional, lumped model of the peripheral vasculature, a method that simplifies the peripheral vasculature into a network of resistors, capacitors, and inductors to approximate pressure and flow dynamics [18]. This approach is particularly useful for shunt design as well as pulmonary and coronary perfusion.

As described, over time, CFD has allowed the optimization of cavopulmonary connections and highlighted the importance of minimizing energy loss and balancing pulmonary flow distribution for improved outcomes. Brief background and recent developments in the Norwood and Fontan procedures are discussed below followed by other notable domains of work within the simulation of single ventricle defects.

#### 4.1.2. Fontan Conduit Optimization and Exploration of Modifications to the Fontan Surgery

Work from de Leval highlighted the importance of energy loss in Fontan hemodynamics, a parameter that may be optimized through vessel size, conduit shape, and distribution of blood flow. Elevated energy loss, often resulting from geometric factors or stenosis within the Fontan pathway, has been associated with decreased systemic flow and cardiac index [19]. These findings suggest that optimizing the distribution of blood flow through adjustments in vessel geometry or surgical intervention may help mitigate energy loss and improve hemodynamic outcomes. Furthermore, the finding that altered hepatic flow distribution (a common finding in the Fontan conduit above the entry of the hepatic venous flow into the inferior vena cava) [20] to the lungs may lead to pulmonary arteriovenous malformation has led to major consideration of hepatic flow distribution in the optimization and study of conduit design [21,22,23]. Recent CFD studies have shown reason to prefer the utilization of Y-graft conduits rather than T-graft conduits in Fontan surgery due to better energy loss and hepatic flow distribution results, likely due to avoiding collision of the SVC and IVC blood flow [24,25]. These results bolster Marsden and colleagues’ previous work that demonstrated, when compared to standard single extracardiac conduits, the Y-graft with 12-mm diameter branches had lower shear stress and greater equilibrium of pulmonary flow [16]. More specifically, compared to diameter-preserving Y-graft conduits (20-10-10 mm where branch flow velocity increases significantly in the beginning), area-preserving Y-graft conduits (20-14-14 mm where the sum of the cross-sectional area of the two branches was approximately equal, resulting in a gradual reduction in flow velocity) led to favorable energy loss results [26]. CFD has also guided the evaluation of novel Y-graft conduits, such as one from Lakshkarinia and colleagues that directs hepatic venous flow to the azygos vein and pulmonary arteries, demonstrating promise as an alternative for complex patients with interrupted inferior vena cava-azygos and hemi-azygos continuation [27]. Conduit size has been of major interest, particularly due to the hypothesis that conduits become undersized over time as the patient grows, leading to adverse clinical outcomes. Utilizing CFD to calculate energy loss, pressure gradient, and normalized resistance during rest and simulated exercise, Rijnberg and colleagues conducted a 3-year prospective growth study that confirms this hypothesis [28]. The group found a mean conduit cross-sectional area 35–73% smaller than patient geometry, leading to suboptimal hemodynamics for conduits sized 16–20 mm. However, as mentioned, since large conduit sizes have been associated with hypoperfusion, it is critical to evaluate current options for conduit sizing (14–22 mm). As such, research has indicated that although somatic growth may interfere with hemodynamics over time, 16–18 mm conduits avoid redundant space (leading to hypoperfusion) and optimize energy loss [29].

Much work has been done to optimize conduit size for the total cavopulmonary connection (TCPC) through CFD. Modifications to the TCPC surgery itself have also been explored through CFD, with recent work evaluating not only modifications to the placement of the extracardiac conduit but also larger changes to create converged venous outflow. Wei et al. explored the possibility of connecting the extracardiac conduit to the anterior surface of pulmonary arteries (PA) or superior vena cava rather than to the inferior PA surface, as in traditional TCPC. Among 25 Fontan patients, similar indexed power loss and hepatic flow distribution values suggest that an anterior PA design can be utilized as an alternative to traditional TCPC and that an SVC design may be beneficial for patients with complex PA anatomy. Rather than change the extracardiac conduit connection point, Sinha et al. explored the idea of creating a new access point within a single inflow/single outflow system to ultimately allow for improved flow efficiency and improved accessibility for the addition of mechanical circulatory support devices [30]. Using CFD, the authors explore the feasibility of this idea, termed the convergent cavopulmonary connection (CCPC), and find that the CCPC configuration had comparable indexed power loss when compared to the traditional TCPC in varying patient sizes. Further optimization is certainly required; however, the development of a novel, promising alternative to the TCPC using CFD is notable and warrants attention as a newer development in the field.

#### 4.1.3. CFD Virtual Planning + Pairing with Other Modalities (4D MRI)

An important note is that CFD studies analyzing hemodynamics of the Fontan sequence are often performed by using averaged clinical data from groups of patients to draw conclusions regarding general energy loss and perfusion. Computational modeling, however, has also proven useful for surgeons on a patient-by-patient basis where an individual CT scan guides a 3D reconstruction and patient-specific clinical values are utilized as boundary conditions. Sundareswaran [31] et al. published the first use of surgical planning for pulmonary arteriovenous malformations; this work was furthered by Haggerty et al. [32] and Trusty et al. [33], who utilized virtual planning and later compared their predictions to post-operative imaging to assess its accuracy. This approach can help assess pre-operative hemodynamic conditions, identify anatomical constraints, and provide acceptable predictions of post-operative hemodynamics. However, it is important to note that, although helpful, utilizing CFD for surgical planning still requires improvements to increase the accuracy of predicted flows to match real-world outcomes, as noted by results published by Trusty et al. A recent paper utilizes this approach in three complex cases with artifact-rich imaging, highlighting the combined use of CT and MR imaging to improve the segmentation of the TCPC anatomy, which could not have been accomplished by either modality separately [34]. Virtual surgical planning has also become critical in better understanding how to optimize the size of conduits, given that they lack growth potential leading to many patients out-growing their conduit and developing non-optimal flow. A recent example is provided by Hut et al., who explore this concept by utilizing CFD to evaluate the impact of virtual conduit expansion on pressure gradients and thrombosis risk on five patient-specific Fontan conduits. The authors conclude, as hypothesized, that larger (24–32 mm) conduits significantly improve hemodynamic efficiency while maintaining low thrombosis risk [35].

Regarding patient-specific Fontan hemodynamics, it is not uncommon to encounter the pairing of CFD with other modalities to achieve more optimized results. A 4D MRI is perhaps the most prevalent, providing quantitative hemodynamic data such as energy loss and pulmonary flow distribution, although at the expense of low spatiotemporal resolution. When paired with CFD and clinical measurement of blood pressure, the hemodynamics of local and systemic vasculature can be quantified more reliably and have contributed to many studies’ mapping of energy loss in Fontan patients. Building on prior work characterizing average hemodynamic metrics for Fontan patients, recent work by Lee et al. utilized a CFD model paired with blood pressure measurements and 4D MRI to determine baseline characterization for post-Fontan infants in particular [36]. The authors report differences between CFD results guided by 4D MRI versus data from 4D MRI alone and conclude that the pulmonary flow distribution values from the conduit as well as viscous dissipation (internal friction value that correlates with decreased exercise capacity and increased liver congestion [37]) values were significantly different between the two methods, with CFD guided by 4D MRI providing greater accuracy due to its higher spatial resolution (0.4 mm vs. 2 mm for 4D MRI alone). Although we highlight the benefits of combining CFD techniques with the advantages of 4D flow MRI, it is crucial to consider the computational expense of experiments—4D MRI involves complex data processing, and high-volume data are not always accessible.

#### 4.1.4. Balancing Feasibility with Expense and Validating CFD for Fontan Application

Although there has been progress made on the Fontan CFD workflow, there is still a way to go for widespread clinical acceptance. The average time for CFD analysis for Fontan surgery is two months [38,39], complex pediatric geometries often result in discrepancies in CFD results, and even if CFD analysis may be possible for surgical planning, there are limited qualified engineering teams to support clinicians to run such analyses. Therefore, optimizing the balance between the efficiency and accuracy of CFD simulations has become critical in the journey toward practical adoption. Recently, the accuracy and efficiency of the current CFD workflow were tested against an in vitro model that utilized a high-resolution pressure sensor to detect indexed power loss [40]. This concept is similar to other studies that utilize 4D flow MRI and particle tracking methods to estimate flow distributions but does not have the same spatial resolution disadvantages and does not necessarily require validation from other simulations [41,42]. As such, the in vitro model may be assumed to be “true values” when compared to the CFD outputs. When comparing solver sophistication and mesh size in the CFD workflow, the authors suggested that there are diminishing returns on investment in accuracy and solving time, with most simulations demonstrating a 60–70% probability of predicting important parameters with >90% accuracy. Kung et al. built on the literature for CFD validation techniques through their introduction of an in vitro mock circuit coupled with a lumped-parameter numerical model. Since the system operates at high fidelity, it can provide closed-loop feedback for CFD studies and enable the creation of a testing environment [43]. As the authors utilize a lumped-parameter numerical model (which solves a smaller set of algebraic or ordinary differential equations in 0D or 1D compared to a 3D numerical solver that uses partial differential equations), they opt for a less demanding computational burden. Although the authors test their model with Fontan graft scenarios, such a framework may enable future work on physically testing medical devices as well. Such results indicate the obvious utility of CFD and provide evidence through novel validation techniques that it provides reliable data when compared to an in vitro model, but more importantly, it underscores the practical usage of current CFD schemas. Frieberg and colleagues build on this point through their “lean” CFD model that provides fast and reliable results for Glenn and Fontan simulations, reducing solution time from about 7 h to just 3 min [44]. The authors combine assumptions that have been previously shown to have an acceptable balance between computation and accuracy, such as rigid vessel walls [45] and laminar flow instead of pulsatile flow [44] with the immersed boundary method (a hexagonal element-based method that requires less user interaction and fewer elements to process) [46,47] to reduce computation time. Workflow changes, such as integrating the CFD solver with the geometrical editing tool, were also reported to play a significant role in the reduction. Rasooli et al. recently provided a performance assessment of another alternative: a computational low-cost Reynolds-averaged Navier–Stokes (RANS) k-ɛ model for the prediction of hepatic flow distribution, power loss, and total pulmonary flow split, finding that the RANS model had 100 times lower computational time and better accuracy when compared to a traditional unsteady laminar solver [48]. The RANS k-ɛ model is an approach that uses time-averaged governing flow equations and adds effects of turbulence, which can be modeled using kinetic energy (k) and turbulent dissipation rate (ɛ)—this process greatly reduces the computational cost and is one of the most widely used models in engineering practice. The authors recognize that although flow dynamics in Fontan studies have previously been considered unsteady laminar, newer work has shown that complex impingement of jet flow streams in the cavopulmonary connection can cause turbulence, which can be modeled through RANS simulation [49]. Although Rasooli et al. found that the RANS model was more efficient and accurate than a traditional unsteady laminar solver, the impact of the k-ɛ model on the RANS model is left to truly be analyzed.

## 5. Aortic Coarctation

Coarctation of the aorta (CoA) accounts for approximately 5–8% of CHDs, with patients experiencing significantly higher long-term morbidity in the forms of early coronary artery disease, hypertension, and heart failure [50]. Such long-term effects were previously hypothesized by O’Rourke in 1971 and Ong in 1972, who suggested that altered hemodynamics in the aorta can impact vascular function [51,52]. Currently, the first-line treatment for CoA is surgery via left thoracotomy to resect or stent the coarctation, with percutaneous methods emerging as a strategy for high-risk neonates.

With CFD advancements—patient-specific modeling, graft sizing and placement, and surgical planning—being successfully applied to studying other CHDs, many began to use CFD to explore the biomechanics of a disease whose hemodynamic instability is implicated in its progression [53]. Using this tool, there is potential to more accurately predict disease progression and ultimately prevent the significant morbidities associated with CoA. We refer the reader to LaDisa et al.’s 2011 review to understand the challenges in modeling the unique CoA geometry [53]. It should be highlighted that CoA demands detailed attention in CFD due to its disruption of aortic compliance, variability in tissue properties, and the influence of small collateral vessels such as the intercostal arteries.

### 5.1. Non-Invasive Methods for CoA Pressure Calculation/Diagnosis

A higher pressure difference through the narrowed aorta (via peak systolic pressure drop (PSPD)) suggests more significant narrowing and impaired blood flow, which can inform the urgency of treatment. Currently, the gold standard of pressure calculation remains cardiac catheterization due to the inaccuracies noted in non-invasive techniques such as MRI or echocardiography [54]. However, with the advent of CFD, many have investigated the potential to pair CFD with other imaging methods to avoid the risks associated with invasive catheterization.

Sotelo et al., in 2015, studied similarities between a CFD-MRI determination of pressure and cardiac catheterization for seven patients and found an acceptable level of agreement. The mean peak-to-peak pressure gradient was 10.36 ± 6.54 mmHg for the catheterization and 9.77 ± 6.39 mmHg for the simulation [55]. A 4D MRI is an alternative to traditional cardiac magnetic resonance (CMR) in that it provides three-dimensional blood flow velocities, which allows for the calculation of dynamic pressure differences. Riessenkampff and colleagues investigated 4D MRI pressure calculation accuracy in patients with CoA and found that pressure fields aligned well with invasive catheter measurements [56]. Despite the advantages of 4D MRI pressure calculation, the method still has notable shortcomings, such as limited spatiotemporal resolution and artifacts [57,58]. In 2022, Shahid and colleagues created a patient-specific, adaptive mesh refinement (AMR)-based CFD method from 4D MRI to address these issues. AMR is a technique that dynamically adjusts the resolution of the mesh based on the complexity of the flow in different regions, resulting in increased computational efficiency and increased resolution of critical flow areas. When comparing their CFD-MRI method with 4D MRI alone, they found clinically acceptable agreement in flow rate values, with significantly improved spatiotemporal resolution in the CFD results [59].

As an alternative to expensive and technically demanding MRIs, Zhu and colleagues studied peak pressure and velocities between a CFD-CTA model to develop evidence for non-invasive pressure measurements for CoA patients. They found agreement (*p* < 0.001) between their CFD model and their invasive catheterization control in predicting peak pressure and velocities in 25 patients [60]. To further refine CT-based CFD studies for CoA, Zhang et al. tested two CFD methods, transverse velocity asymmetry (TVA) and transverse velocity fraction (TFA), in estimating critical pressure drops for 40 patients. TVA quantifies the unevenness of blood flow velocity across a vessel’s cross-section, helping assess flow distribution, while TVF measures the proportion of total blood flow occurring transversely, indicating non-axial flow patterns. When comparing the two CFD methods to invasive catheterization pressure values, the authors found that the TVA strategy had the strongest correlation (r = 0.93) with catheter values compared to TVF (r = 0.83) or echocardiography (0.67) and the strongest sensitivity at 0.92 [61].

Recent studies have highlighted the need to define novel methods that do not require invasive measurements to set boundary conditions (i.e., eliminate the need to perform catheterization to accurately set parameters for the CFD simulation). Aslan et al. address this need in traditional CMR by analyzing the performance of a CFD model based on boundary conditions set through non-invasive blood pressure measurements [62]. When validating this method against invasive catheterization, the authors found that the CFD model had clinically similar aortic PSPD measurements. However, their blood pressure measurements were not taken during patient sedation and thus caused discrepancies between the CFD-predicted and invasively measured results by 5.5 mmHg or less. The 4D MRI methods, such as the one described by Riessenkampff [56], typically require validation against other invasive tests. In 2019, Saitta et al. described a process in which 4D MRI values can be validated against a fluid/structure interaction (FSI) model in the context of CoA, essentially eliminating the need for any other imaging or measurement method [63]. By testing 4D MRI results with a simulation based on the 4D flow data, the authors demonstrate a non-invasive validation aspect of 4D MRI that can potentially accelerate the clinical adoption of 4D MRI fluid flow analysis as an alternative to catheterization. Similarly, Lu and colleagues developed a CFD method based on CTA imaging alone. The authors trained this method on 52 CoA patients’ PSPD values to eventually use it to classify patients into CoA or non-CoA groups. When testing this model, the authors revealed a high diagnostic performance (average AUC = 0.958) when run against a testing set of 13 CoA patients. The authors then tested the ESC (European Society of Cardiology) non-invasive criteria for CoA intervention and found that these criteria performed poorly in the dataset, indicating that, despite the study’s small sample size and basic boundary conditions, solely relying on guidelines may lead to underdiagnosis and treatment for patients who may benefit.

Fetal echocardiography is a widely used method that may enable more widespread utilization of CFD to understand CoA. Spatiotemporal image correlation (STIC) in echocardiography combined with a special mode called tomographic ultrasound imaging (TUI) can create images in a series of slices in x, y, and z planes, much like slices of a CT scan. Chen and colleagues utilized TUI in echocardiography to create a CFD model to investigate five CoA patients to determine a threshold for hemodynamically significant CoA. The authors found that a 55% reduction of the aortic isthmus led to an exponential change in velocity, pressure, and WSS. The widespread availability of echocardiography over MRI has an important implication in the context of CFD in low- and middle-income countries (LMICs), as highlighted by Swanson and colleagues in their development of a resource-efficient CFD pipeline for LMICs [64]. Swanson et al. highlight the importance of using open-source software and echocardiography to increase accessibility. Despite the gap in accuracy between MRI-based and echocardiography-based CFD, the research team argues that this pipeline demonstrates that low-resource pipelines are possible and that more work to improve the accuracy of echocardiography-based CFD can further accessibility for LMICs.

Despite progress in non-invasive diagnosis and classification of CoA, most studies are limited by small sample sizes and boundary conditions that do not perfectly align with a patient’s real clinical presentation. CFD presents an opportunity to move toward a quicker, more accurate, and resource-efficient analysis of CoA pressure differentials, but more work is needed to translate these methods into clinical practice.

### 5.2. Stenting for CoA

Since 1991, the clinical application of stenting for CoA patients has been shown to reduce pressure gradients across the narrowing. Despite the reduction in pressure gradient, stents have been shown to alter hemodynamics across the aorta, particularly 3D shear stress distributions [65]. As mentioned earlier, such minute differences in blood flow through a repaired CoA may be responsible for the deleterious long-term morbidities associated with the condition. Other complications of stent placement can also include occlusion of the subclavian artery as well as an increased risk of aneurysm or aortic dissection [66]. CFD has been utilized frequently in CoA research for its evaluation of stenting, whether it be pre- or post-operatively, to prevent complications.

Kwon and colleagues created the first patient-specific CFD model of a patient treated for CoA by stenting [67]. The group compared wall shear stress (WSS) distributions of their implanted Palmaz stenting to two other commonly used stents that were placed virtually, the Cheatham Platinum (Hopkinton, NY, USA) stent and the Genesis XD (Cordis Corp., Cardinal Health, Dublin, OH 43017, USA) stent. The authors found that the Genesis XD model had the least turbulent flow, with their results laying down a workflow for future studies aiming to evaluate stenting for CoA through CFD.

This study and many others utilize a common modeling method called the finite element method (FEM), which allows for WSS calculation at individual struts on the stent [68]. Despite this level of detail, there is a large computational cost and lack of quick customizability once results are generated, leading to increased use of time and resources. This, paired with the potential to quickly simulate placement in the assistance of pre-operative planning, began the traction for the development of virtual stent placement environments, first seen in intracranial stenting [68,69]. By focusing on the periodicity of the stent mesh, these methods create a model of the aorta that is computationally malleable in which deformations resulting from stent placement can be quickly applied to a simulation. In 2016, Neugebauer and colleagues applied findings in virtual stent placement to create a stent placement set-up based on medical guidelines whose parameters can be altered by a user [67]. Although this was the first instance of virtual stent placement for CoA that allowed for the deformation of the whole aortic mesh, the authors indicate that interpersonal variability, particularly at the transition zone between the vessel and stent, was a major limitation. Despite this, Neubebauer’s work, along with more recent studies from Chen et al. (2018) and Kan et al. (2021), have demonstrated a validated protocol for in silico modeling for CoA [70,71].

In addition to virtual modeling protocol, however, accurate simulation of the effect of stenting on hemodynamics is also critical. Foundational work has established the importance of spatiotemporal differences in laminar and turbulent flow in the heart. Three CFD simulation methods—Reynolds-averaged Navier–Stokes equations (RANS), large eddy simulation (LES), and direct numerical simulation (DNS)—are typically considered for turbulence modeling, with accuracy and computational expense increasing accordingly [72]. The aforementioned CFD techniques are based on solving the Navier–Stokes governing equations for fluid flow. In the last decade, the lattice Boltzmann (LB) method has gained popularity in studies of turbulent flow due to its simpler, more efficient equation-solving approach, which effectively handles complex geometries. This advantage makes it an attractive alternative to traditional methods for simulating fluid dynamics in intricate systems [73]. Previous studies have applied the LB method to CoA, finding that this method can produce realistic pressure values [74,75,76]. Using this groundwork, Dandan and colleagues developed a framework that uses the LB numerical approach with the LES turbulence modeling method to assist in silico stent placement for CoA [72]. They demonstrated that this CFD method, paired with existing virtual stenting techniques, as mentioned earlier, can accurately predict aortic flow with acceptable computational cost and assist with deciding on an optimal stent diameter when accompanied by MRI imaging. By implementing CFD methods proven to improve the accuracy and efficiency of CoA simulation into an existing modeling framework, the authors demonstrate a robust new framework for in silico stent placement.

A major limitation of these in silico stent placement methods, particularly those relying on simplified modeling approaches, is the lack of detailed interaction between aortic geometry, material properties, and stent behavior. Unlike more comprehensive methods like FEM, which can account for these intricate interactions, simplified models may not accurately predict the deformed aorta post-stenting.

Finally, alternative approaches to traditional stenting techniques, such as tissue-engineered vascular graft (TEVG) placement and bypass surgery for CoA, have benefited from advancements in CFD. Liu et al. developed a framework to automatically optimize TEVG shape through a model by first modeling deformation through free-form deformation, a method that warps the space around the geometry, then optimizing shape through Gaussian process regression, and lastly simulating flow through CFD utilizing a Navier–Stokes solving method [77]. The authors demonstrated that their model produced optimal energy loss values from simulation but demonstrated that the coarctation in one of their models was not resolved, likely due to restriction in their shape optimization equation. Surgical bypass is sometimes performed over stenting or anastomosis when recurrent coarctation with restenosis, severe arch hypoplasia, or extensive aortic tortuosity or calcification. Fujisue and colleagues employed a Navier–Stokes-based CFD model to compare various bypass routes for two complex CoA patients aged 76 and 68, with the optimal route employed during both surgical repairs [78]. The authors suggest that since CFD does not provide information on peripheral arterial resistance and the reaction of baroreceptors and endocrinology, surgeons should collect additional information before operation. An evaluation of bypass grafting through an LB-based CFD approach was performed in 2022 by Sadeghi and colleagues [79]. In a study on three patients’ CoA patient hemodynamics pre- and post-extra anatomical bypass surgery, the authors found that grafting may lead to pseudoaneurysm formation, potential aortic rupture, and higher shear stress that can lead to deleterious remodeling despite modest improvements in overall shear stresses and flow velocities.

### 5.3. Special Morphologies

After CoA repair, aortic arch remodeling may occur with implications for long-term hemodynamics and clinical outcomes. Gothic (sharply angled arch) geometries have been shown to have higher resting hypertension as well as exercise-induced hypertension, with long-term studies indicating that this hemodynamic instability may lead to aortic stiffness [80]. Romanesque (well-rounded arch) geometries, on the other hand, are indicative of more favorable hemodynamics. Recently, work has focused on utilizing CFD to analyze not only the prevalence of Gothic geometries in patients but also the spatiotemporal distribution of stresses that lead to hemodynamics risk. In 2022, Zhang and colleagues explored the latter, finding that Gothic geometries display high velocity, specifically toward the inner wall around the arch apex throughout the systolic phases [81]. The authors also reported that Gothic geometries had more temporal and spatial variations of wall shear stress in the descending aorta. Qin et al. studied both prevalence and hemodynamics in 2023, finding that the Gothic arch is common, has a higher aortic arch height-to-width ratio, and represents significantly higher ascending to descending aorta angles than other geometries [82]. More research is indicated in this field, particularly to determine what pre-operative hemodynamic or geometric conditions may predispose patients to the Gothic arch geometry.

### 5.4. Machine Learning for CoA

Despite the vast literature surrounding CFD and its application to CoA and other CHDs, the inherent complications of CFD, such as highly specialized programming, time and resource expenditure, and concerns regarding validation, remain. With the advent of machine learning, researchers have begun to explore its applications in hemodynamic modeling as a cost-effective, quicker alternative to CFD for aortic hemodynamics [83]. Concerns regarding model accuracy, lack of expansive training sets, and minimal studies with practical application drove Yevtushenko and colleagues to perform a proof-of-concept study in which they created a deep artificial neural network capable of computing hemodynamics for CoA patients [84]. Through this neural network trained on an extensive dataset of CoA geometries produced through CFD, the authors explain that this technology can produce results almost instantly on any type of hardware. They compared their machine learning results with CFD and found that the neural network performed well on the majority of test cases and predicted centerline aortic hemodynamics particularly well. The key shortcoming, however, is that the ground truth of the neural network was CFD-based results, meaning that the neural network accuracy is only as good as the accuracy of the CFD model used to train the network. Neural networks are dependent on the data they are trained on, meaning their predictive accuracy relies on the similarity of new data to the distribution of the training data. Thus, if the training data are flawed, the network will inherently learn these inaccuracies, leading to erroneous predictions. Furthermore, there were several cases of large pressure discrepancies in the results. Despite this, it is clear that there is potential for a new avenue of hemodynamic research for CHD, one that produces results quicker than ever before.

## 6. Conclusions

The evolution of CFD has established it as a highly valuable tool in the analysis and treatment of cardiovascular diseases. It is important to note that CFD results are only as accurate as the model in which the simulation is run, meaning the simulation is only as accurate as the boundary conditions and assumptions that were made to create that model in the first place. This accentuates the reliance on multiple variables and decision-making processes within the CFD workflow, regardless of novel methods that have been developed. Despite this, CFD models have shown utility and promise for translation to the clinic and much work is underway to improve the accuracy of such models through more robust computational methods. Furthermore, the growing demand for patient-specific cardiovascular techniques underscores the need for clinicians and researchers to understand the applications and limitations of CFD.

With this in mind, our review first describes the basics of CFD by outlining the traditional CFD workflow and then discusses CFD applications in the most relevant congenital heart disease fields. Here, we demonstrate CFD’s capability to offer an understanding of complex cardiac morphology as seen in HLHS and uniquely position surgeons to supplement clinical decision-making with quantitative data. We explore the applications of CFD in recent advances in medical device solutions for congenital heart disease. Finally, we review the iterative nature of improvement in CFD techniques and offer insight from the literature on how to balance the costs of simulation with model accuracy.

## Figures and Tables

**Table 1:** CFD Terms and Definitions.

Term	Definition
Pressure	The amount of force per unit surface area that acts on a surface in the direction perpendicular to the surface.
Velocity	The speed and direction of the fluid (i.e., blood) inside or outside the model.
Flow Rate	The volume of a fluid passing through per unit time.
Energy Loss	The dissipation of mechanical energy within a fluid flow system, typically due to friction against boundaries or internal turbulence, converting this energy into heat and reducing the total available energy within the flow.
Wall Shear Stress (WSS)	The shear force produced by tangential blood flow on the vessel wall as a result of blood viscosity. Related to the gradient of velocity in the surface normal direction.
